# Pneumopericarditis: A Case of Acute Chest Pain with ST Segment Elevation

**DOI:** 10.1155/2015/256546

**Published:** 2015-06-14

**Authors:** Erwin E. Argueta, Menfil A. Orellana-Barrios, Teerapat Nantsupawat, Alvaro Rosales, Scott Shurmur

**Affiliations:** ^1^Department of Internal Medicine, Texas Tech University, Lubbock, TX 79430, USA; ^2^Department of Cardiovascular Medicine, Texas Tech University, Lubbock, TX 79430, USA

## Abstract

Pneumopericarditis describes a clinical scenario where fluid and air are found within the pericardial space. Although infrequent, pneumopericarditis should be considered in patients presenting with acute chest pain as a differential diagnosis. This is relevant in patients with history of upper gastrointestinal (GI) surgery, as this may lead to a fistula communicating the GI tract and the pericardium. We report a 42-year-old man with history of numerous surgical interventions related to a Nissen fundoplication that presented with acute chest pain and inferior lead ST segment elevations. Emergent coronary angiography was negative for coronary vascular disease but fluoroscopy revealed air in the pericardial space. Subsequent radiographic studies helped confirm air in the pericardial space with a fistulous communication to the stomach. Ultimate treatment for this defect was surgical closure.

## 1. Introduction

Pneumopericarditis involves a pericarditis clinical presentation, where fluid and air are found within the pericardial space. There are many reported cases of patients with a pneumopericardium, with only a few developing a classical pericarditis syndrome. This last condition has been described most frequently secondary to cardiovascular surgery, trauma, and gastrointestinal (GI) cancer. It may also result from abdominal surgery related complications, where the GI tract forms a fistula to the pericardial space. The latter is also known as a gastropericardial or esophagopericardial fistula depending on the organ involved. The pneumopericarditis clinical presentation varies from acute chest pain and hemodynamic compromise to a subacute and even chronic pericarditis picture. We present a patient with acute pneumopericarditis that had history of a Nissen fundoplication 14 years before with multiple postoperative complications requiring several interventions.

## 2. Case Presentation

A 42-year-old man, active smoker, presented to the emergency room complaining of anterior left side chest pain that started approximately three hours before arrival. He woke up around 3:00 am with sudden onset severe crushing chest pain that was constant. It was associated with shortness of breath and diaphoresis. His medical history was significant for gastroesophageal reflux, hypertension, major depression, and chronic left shoulder pain diagnosed as impingement syndrome. Family history was noncontributory. He had an extensive past surgical history, which included a laparoscopic Nissen fundoplication at age 25 due to severe gastroesophageal reflux disease and associated esophagitis. Several months after this procedure, he required two open revisions of the Nissen procedure. The first was due to a hiatal hernia, and the second was due to a diaphragmatic rupture requiring diaphragm repair with prosthetic material. The patient developed a subdiaphragmatic abscess at age 30 that required antibiotics and diaphragmatic surgery where the prosthetic material was removed. In addition, at age 35 years, he had a spinal cord stimulator inserted, as treatment for chronic left shoulder and epigastric pain associated with persistent nausea and dry heaves. Three months prior to his current visit he had a diagnostic arthroscopy due to persistent left shoulder pain.

**Figure 1:**
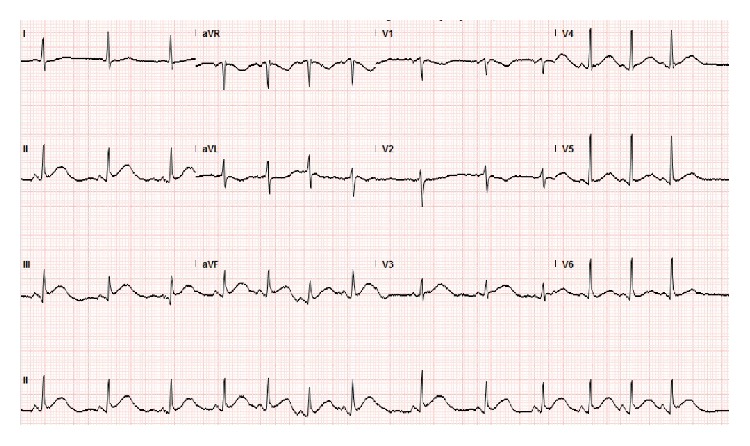
Admission EKG. ST segment elevations are noted in leads II, III, and aVF.

**Figure 2:**
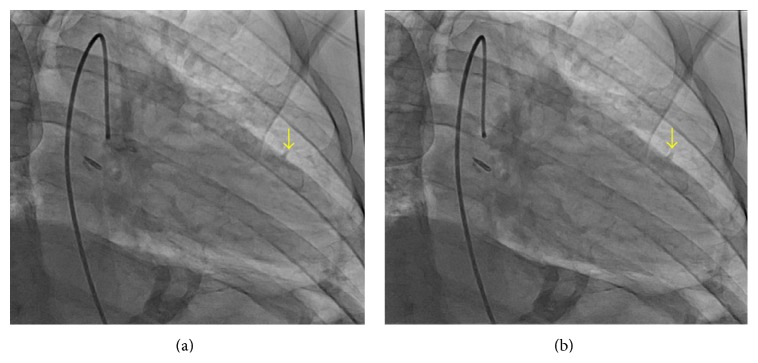
Left ventriculogram. Air around the cardiac borders noted in systole (a) and diastole (b).

On physical exam, he was pale and in distress. Vital sings included an oral temperature of 97.6°F, blood pressure of 116/66 mmHg, heart rate of 45 beats per minute, respiratory rate of 18 breaths per minute, and oxygen saturation of 94% on room air. No rub or murmur was auscultated on initial cardiac examination. Lungs were clear to auscultation. The electrocardiogram (EKG) was significant for ST segment elevations in leads II, III, and aVF (Figure 1). TIMI risk score was 2 (based on chest pain and EKG changes). The patient was immediately taken for coronary angiography, which showed no coronary artery obstruction. However, a halo around the cardiac silhouette suggestive of air was noted (Figure 2). Laboratory results came back during the procedure and were significant for a white blood cell count of 15.42 K/*μ*L (88.5% neutrophils), creatinine of 1.4 mg/dL, and glucose of 172 mg/dL. Troponin T was negative and the rest of the laboratory values were noncontributory.

**Figure 3:**
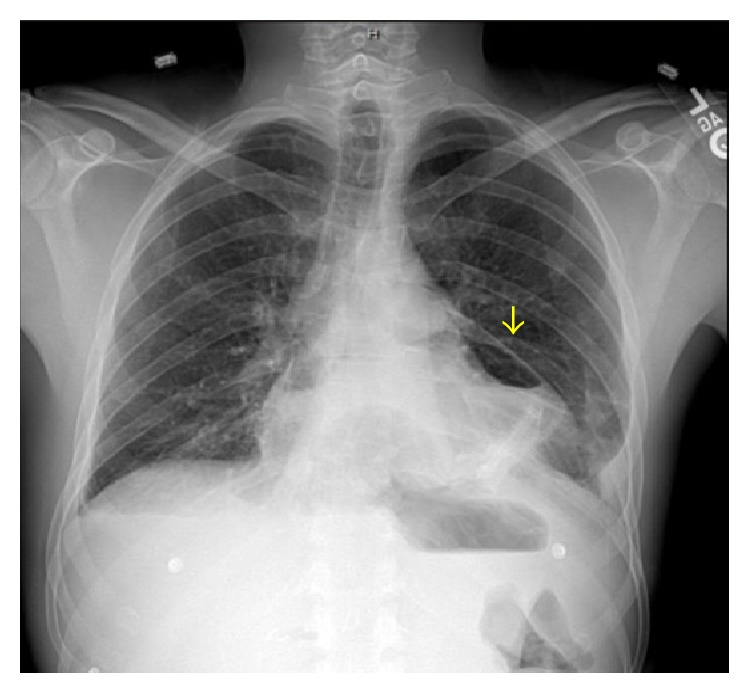
PA chest X-ray. A halo is visible around the left cardiac border.

**Figure 4:**
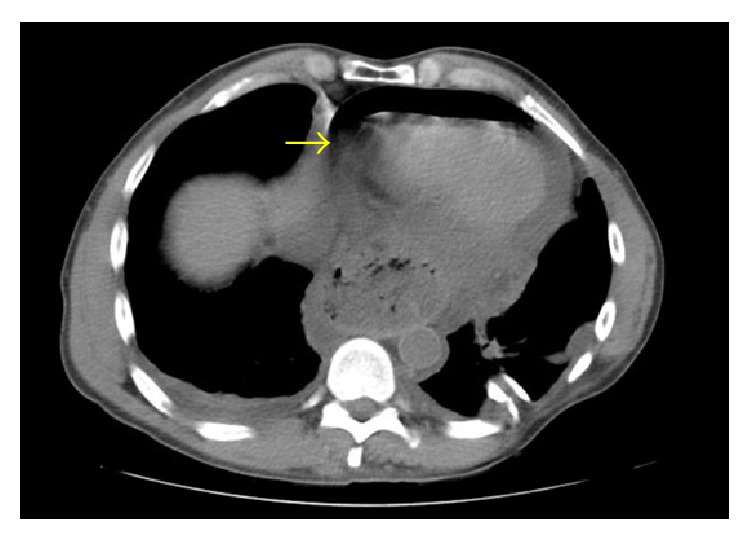
Chest computed tomography. There is an air fluid level in the pericardial space.

**Figure 5:**
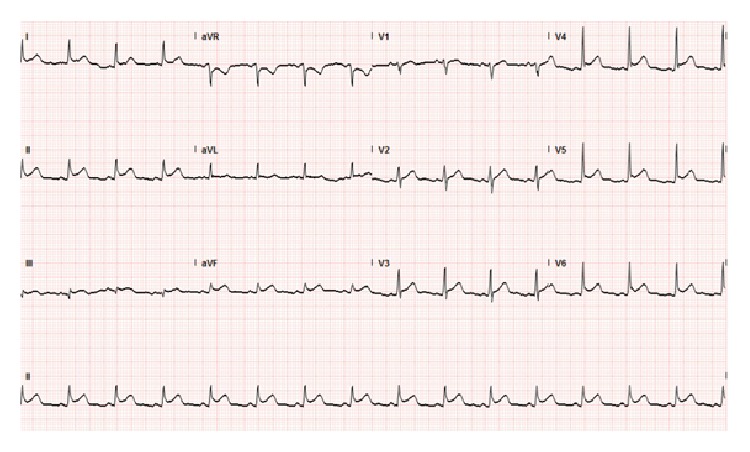
EKG 24 hrs after admission. Diffuse ST segment elevation and slight PR segment depression.

**Figure 6:**
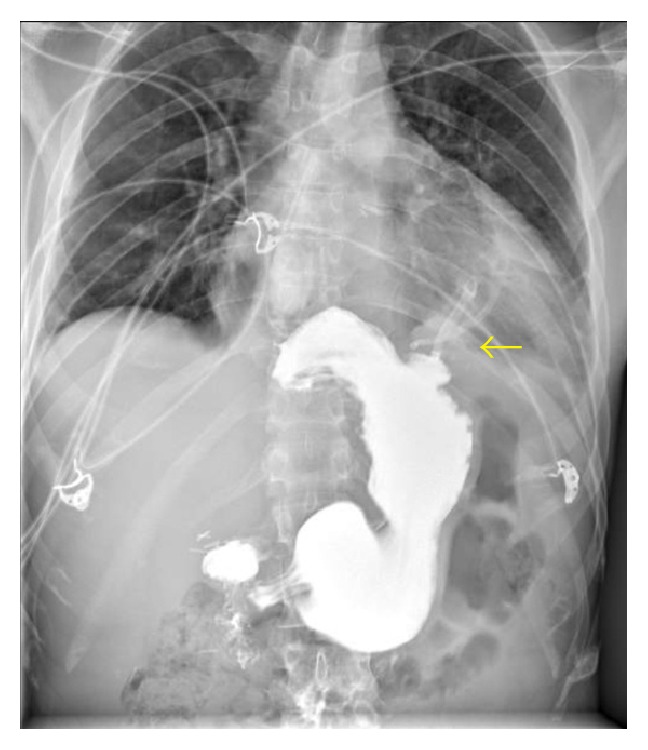
Contrast esophagogram. There is evidence that suggests a fistula located at the level of the cardia.

A chest X-ray obtained after the angiogram also showed this halo around the left cardiac border suggesting a pneumopericardium (Figure 3). A chest computer tomography confirmed the diagnosis (Figure 4). During the following 12 hours while additional diagnostic workup was performed, the patient developed signs consistent with acute pericarditis. This included a mill wheel type murmur and diffuse ST segment elevations on the EKG (Figure 5). No pulsus paradoxus was detected. A transthoracic echocardiogram performed had technically limited images but was suggestive of a preserved ejection fraction and a pericardial effusion. A Gastrografin esophagogram suggested a pericardial fistula located at the level of the greater curvature of the cardia (Figure 6). After diagnostic workup, the patient was started on empiric antibiotic coverage with meropenem and was taken to the operating room. There he had a pericardial window and upper gastrointestinal endoscopy. About 300 mL of serosanguineous fluid was drained from the pericardial space. During endoscopy, an ulcer was noted at the level of the cardia with no obvious communication to the pericardium. Due to the high suspicion of a communication between these two structures, final treatment consisted in application of a fibrin sealant to the ulcer. Drains were also left in the pericardium and removed later. A pericardial biopsy was consistent with fibrinopurulent pericarditis and a biopsy obtained from the gastric ulcer was negative for* H. pylori* and malignancy. The patient tolerated the surgery well and no organisms were recovered from the cultures obtained from the pericardial fluid.

## 3. Discussion

There have been numerous pneumopericardium case reports secondary to various etiologies, with fistula formation occurring in up to 30% of cases [1, 2]. From this last group, those that arise from the GI tract are associated with gastroesophageal trauma and/or surgery. They can occur after trauma or during the first year after surgery or years after [3, 4]. These fistulae occur secondary to complications from esophagectomy with gastric tube repair, gastric bypass surgery, and slipped Nissen fundoplications. They are found less frequently secondary to spontaneous gastric ulcer disease or gastrointestinal cancer [5–8]. There has also been an association between subphrenic abscesses with repeated gastroesophageal surgeries and fistula development [9]. Out of the patients with pneumopericardium secondary to fistula formation, the minority will develop an acute pericarditis clinical picture.

The common symptoms described by patients with pneumopericardium are chest pain and dyspnea. Left shoulder pain, or referred pain, may also be present and arises from diaphragmatic and/or pericardial irritation [10–12]. Of note, in some cases shoulder pain is chronic and persistent. On physical examination a classic mill wheel murmur, or “bruit de moulin,” can be present, which is a metallic tinkling rub found in patients with pneumopericardium [1]. Diagnosis is based on imaging studies which include upright chest X-rays, echocardiography, and chest computed tomography. GI imaging studies, like contrast esophagography, may show a fistula to the pericardium. Electrocardiographic findings can vary from those consistent with pericarditis to those with localized ST segment elevations. For example, much like in the case presented, these electrocardiographic findings have led to angiography and thrombolysis (Table 1) [2, 8, 9, 13, 14]. Treatment includes conservative management, described in few cases, and surgical intervention with fistula correction. Cases with conservative management have a worse prognosis, with the mortality being as high as 85% [8, 15]. A case has been described where the fistula was unrepaired and the patient had recurrent disease five years later [16].

In our case, the patient may have had chronic diaphragmatic irritation leading to his chronic left shoulder pain. It is difficult to say over what period of time he started to have pericardial irritation with a fistula formation. A possible explanation for the absence of clinical signs of tamponade is that the fistula could have a bidirectional flow thereby impeding higher pressure buildup in the pericardial space over time. In this sense, a possible acute decompensation with increase in symptoms may have been due to changes in the fistula flow or gastric content/pressure leading to his acute symptoms and hospital visit. This would also be supported by his clinical course while in the hospital. We present electrocardiographic changes that were present after admission in Figure 5. Other cases where EKG changes were not suggestive of pericarditis are shown in Table 1. In patients with risk factors for developing gastropericardial fistulas, chest pain, and ST segment elevations we consider that a chest X-ray performed during the initial assessment should be considered.

In conclusion, pneumopericarditis should be considered as a differential diagnosis for acute chest pain in the patient with extensive gastroesophageal surgery. A key clinical finding such as chronic left shoulder pain and history of multiple GI interventions should heighten the suspicion for a possible gastropericardial fistula. In addition, associated EKG findings should be evaluated with caution in this clinical setting. In cases such as this one, a portable chest X-ray on admission may change a patient's management and avoid unnecessary interventions.

## Figures and Tables

**Table 1:** Pneumopericardium case reports where EKG ST segment elevations were found during initial presentation.

Author	Gender	Age	Risk factor	Chest X-ray^*∗*^	ST elevation^†^	Intervention
Kato et al. [8]	M	65	Esophagectomy	Yes	V5-V6	None
Bruhl et al. [2]	M	63	Spontaneous	No	I, II, aVL, V3–V6	Angiogram
Ruano Poblador et al. [15]	M	56	Billroth I gastrectomy	Yes	Lower, lateral	None
Gagné et al. [13]	F	43	Roux-y-gastric bypass	Yes	I, II, aVL	Angiogram
Sihvo et al. [9]	M	54	Nissen Fundoplication	No	Inferior	Thrombolysis
Grandhi et al. [14]	M	29	Diaphragmatic hernia repair	Yes	V1, V2	None

^*∗*^Chest X-rays usually obtained when there were other exam findings such as fever.

^†^Per case report description.

## References

[1] Brander L., Ramsay D., Dreier D., Peter M., Graeni R. (2002). Continuous left hemidiaphragm sign revisited: a case of spontaneous pneumopericardium and literature review. *Heart*.

[2] Bruhl S. R., Lanka K., Colyer W. R. (2009). Pneumopericardial tamponade resulting from a spontaneous gastropericardial fistula. *Catheterization and Cardiovascular Interventions*.

[3] Murthy S., Looney J., Jaklitsch M. T. (2002). Gastropericardial fistula after laparoscopic surgery for reflux disease. *The New England Journal of Medicine*.

[4] Farjah F., Komanapalli C. B., Shen I., Sukumar M. S. (2005). Gastropericardial fistula and *Candida kruzei* pericarditis following laparoscopic nissen fundoplication (gastropericardial fistula). *Thoracic and Cardiovascular Surgeon*.

[5] Reicher J. J., Mindelzun R. (2014). Case report: benign gastric ulcer erosion leading to a gastropericardial fistula in a patient with no known risk factors. *Clinical Imaging*.

[6] Marasca F. A., Alves G. R. T., Pires R. C., Dallasta T. C., De Andrade Silva R. V., Missel Corrêa J. R. (2013). Gastropericardial fistula. *Annals of Thoracic Surgery*.

[7] Rodriguez D., Heller M. T. (2013). Pneumopericardium due to gastropericardial fistula: a delayed, rare complication of gastric bypass surgery. *Emergency Radiology*.

[8] Kato T., Mori T., Niibori K. (2010). A case of gastropericardial fistula of a gastric tube after esophagectomy: a case report and review. *World Journal of Emergency Surgery*.

[9] Sihvo E. I. T., Räsänen J. V., Hynninen M., Rantanen T. K., Salo J. A. (2006). Gastropericardial fistula, purulent pericarditis, and cardiac tamponade after laparoscopic nissen fundoplication. *Annals of Thoracic Surgery*.

[10] Kim W. J., Choi E. J., Oh Y.-W., Kim K. T., Kim C. W. (2011). Gastropericardial fistula-induced pyopneumopericardium after esophagectomy with esophagogastrectomy. *Annals of Thoracic Surgery*.

[11] Park S., Kim J.-H., Lee Y. C., Chung J. B. (2010). *Gastropericardial fistula* as a complication in a refractory gastric ulcer after esophagogastrostomy with gastric pull-up. *Yonsei Medical Journal*.

[12] Pop D., Venissac N., Rami L., Mouroux J. (2007). Gastropericardial fistula after laparoscopic surgery for gastroesophageal reflux disease. *Journal of Thoracic and Cardiovascular Surgery*.

[13] Gagné D. J., Papasavas P. K., Birdas T., Lamb J., Caushaj P. F. (2006). Gastropericardial fistula after Roux-en-Y gastric bypass: a case report. *Surgery for Obesity and Related Diseases*.

[14] Grandhi T. M., Rawlings D., Morran C. G. (2004). Gastropericardial fistula: a case report and review of literature. *Emergency Medicine Journal*.

[15] Ruano Poblador A., Gay Fernández A. M., García Martínez M. T. (2007). Pneumopericardium caused by gastropericardial fistula. *Revista Espanola de Enfermedades Digestivas*.

[16] Servais E. L., Stiles B. M., Spector J. A., Altorki N. K., Port J. L. (2012). Gastropericardial fistula: a late complication of esophageal reconstruction. *Annals of Thoracic Surgery*.

